# Navigating the complex path between the oxytocin receptor gene (*OXTR*) and cooperation: an endophenotype approach

**DOI:** 10.3389/fnhum.2013.00801

**Published:** 2013-11-28

**Authors:** Brian W. Haas, Ian W. Anderson, Jessica M. Smith

**Affiliations:** ^1^Department of Psychology, University of GeorgiaAthens, GA, USA; ^2^Interdisciplinary Neuroscience Graduate Program, University of GeorgiaAthens, GA, USA

**Keywords:** *OXTR*, genetics, oxytocin, social-cognition, cooperation

## Abstract

Although cooperation represents a core facet of human social behavior there exists considerable variability across people in terms of the tendency to cooperate. One factor that may contribute to individual differences in cooperation is a key gene within the oxytocin (OT) system, the OT reception gene (*OXTR*). In this article, we aim to bridge the gap between the *OXTR* gene and cooperation by using an endophenotype approach. We present evidence that the association between the *OXTR* gene and cooperation may in part be due to how the *OXTR* gene affects brain systems involved in emotion recognition, empathy/theory of mind, social communication and social reward seeking. There is evidence that the *OXTR* gene is associated with the functional anatomy of the amygdala, visual cortex (VC), anterior cingulate and superior temporal gyrus (STG). However, it is currently unknown how the *OXTR* gene may be linked to the functional anatomy of other relevant brain regions that include the fusiform gyrus (FG), superior temporal sulcus (STS), ventromedial prefrontal cortex (VMPFC), temporoparietal junction (TPJ) and nucleus accumbens (NAcc). We conclude by highlighting potential future research directions that may elucidate the path between *OXTR* and complex behaviors such as cooperation.

## Introduction

Human beings have a unique affinity towards cooperating with one another to accomplish goals. The act of cooperation involves a common effort within a group for the collective benefit rather than seeking to accomplish goals solely for oneself. Cooperative people are characterized as socially tolerant, empathic, helpful, and compassionate (Cloninger et al., 1993). Although the tendency to cooperate is a common attribute across many people and cultures, there also exists considerable variability between people in cooperative motivation and behavior (Wischniewski et al., 2009). In this article, we highlight recent progress towards identifying genetic factors that contribute to individual differences in cooperation. Within this framework, we focus our attention on the oxytocin (OT) system and brain regions that subserve the tendency to cooperate.

Cooperation has been empirically investigated in several different ways. One common way that cooperation has been studied within social psychology and economic research is to use the Prisoner’s Dilemma task. The prisoner’s dilemma task provides the opportunity to characterize the value one attributes to their own outcome versus the value one attributes to the outcome of the entire group. Results of this research demonstrate that humans often act more cooperatively than a strict self-interest strategy would predict (Komorita and Parks, 1999).

Although many humans tend to be driven towards cooperation, there also exists considerable variability among people in how cooperative they tend to be (Wischniewski et al., 2009). For example, individual differences in personality traits, such as with agreeableness and conscientiousness, are associated with the tendency to cooperate (Witt et al., 2002; Volk et al., 2011). Another factor that may influence cooperation across humans is genetics (Cesarini et al., 2008). Many of the genes implicated in cooperation are known to affect the function of hormonal and neurotransmitter systems within a network of brain regions important for social-cognition.

One hormone associated with individual differences in cooperation and with the function of brain regions involved in social-cognition is OT (Ebstein et al., 2012; Yamasue, 2013). OT is a neuropeptide primarily synthesized in the hypothalamus and has broad effects on OT receptors throughout the central nervous system (Gimpl and Fahrenholz, 2001). One way to investigate the effect of OT on social behavior is to manipulate OT levels via intranasal administration. Following OT administration people display greater amounts of cooperative behavior as compared to individuals receiving placebo (Kosfeld et al., 2005; Declerck et al., 2013). These findings have motivated the search for genes within the OT system that are linked to individual differences in cooperation.

One gene within the OT system that has been linked to prosocial behaviors, such as cooperation, is the OT receptor gene (*OXTR*). *OXTR* is a gene located on chromosome 3p25 that codes for OT receptors (Kimura et al., 1992). There are several single nucleotide polymorphisms (SNPs) of *OXTR*, each of which codes for particular attributes of the OT receptor. Studying how behavioral or biological metrics vary according to *OXTR* SNPs provides insight as to the function of the *OXTR* gene. In this article, we focus on findings comparing social cognitive metrics, as related to cooperation, based on *OXTR* SNPs.

Behavioral genetic studies associating polymorphisms of the *OXTR* gene with laboratory measures of cooperation have revealed a mixed group of results. Israel et al. (2009) and Tabak et al. (2013) demonstrated an association between *OXTR* and lab measures of cooperation including the Dictator Game and the Social Values Orientation and Prisoner’s Dilemma tasks. On the other hand, Apicella et al. (2010) reported no association between *OXTR* and cooperation during the Dictator and Trust Game. The inconsistency across these studies may indicate that *OXTR* is associated with some, but not all, of the underlying components of cooperative behavior in humans.

One effective method used to investigate the association between genes and complex behaviors, such as cooperation, is to use an endophenotype approach. An endophenotype represents an intermediate level between gene expression and a complex behavior or disease state (Gottesman and Gould, 2003). For example, there may be a weak or moderate association between a gene and the onset of an anxiety or mood disorder based on how the gene codes for the organization of neurons that react to psychosocial stress. In this example, the neuronal response to psychosocial stress is considered an endophenotype that exists between the gene and disease (Hamer, 2002; Gottesman and Gould, 2003). Considering endophenotypes holds promise in terms of elucidating the genetic etiology of cognition, social behavior and psychopathology.

## A component view of cooperation and the cooperative brain

The ability to cooperate effectively with others relies on a set of underlying social-cognitive constructs (Brosnan et al., 2010). Successful cooperation requires social cognitive constructs that include (but not limited to) to (i) acknowledging and recognizing the emotional states of others within a group (emotion recognition) (Elfenbein et al., 2007; Krumhuber et al., 2007); (ii) accurately interpreting the intentions of others (empathy/theory of mind) (Sally and Hill, 2006; Paal and Bereczkei, 2007); (iii) communicating effectively with others (social communication) (Miller et al., 2002); and (iv) seeking out and valuing social interaction (social reward seeking). Thus, effective cooperation may be characterized by the availability of specific social-cognitive resources. Considering each of these social cognitive constructs independently may elucidate how genes within the OT system influence cooperative motivation and behavior in humans.

**Figure 1:**
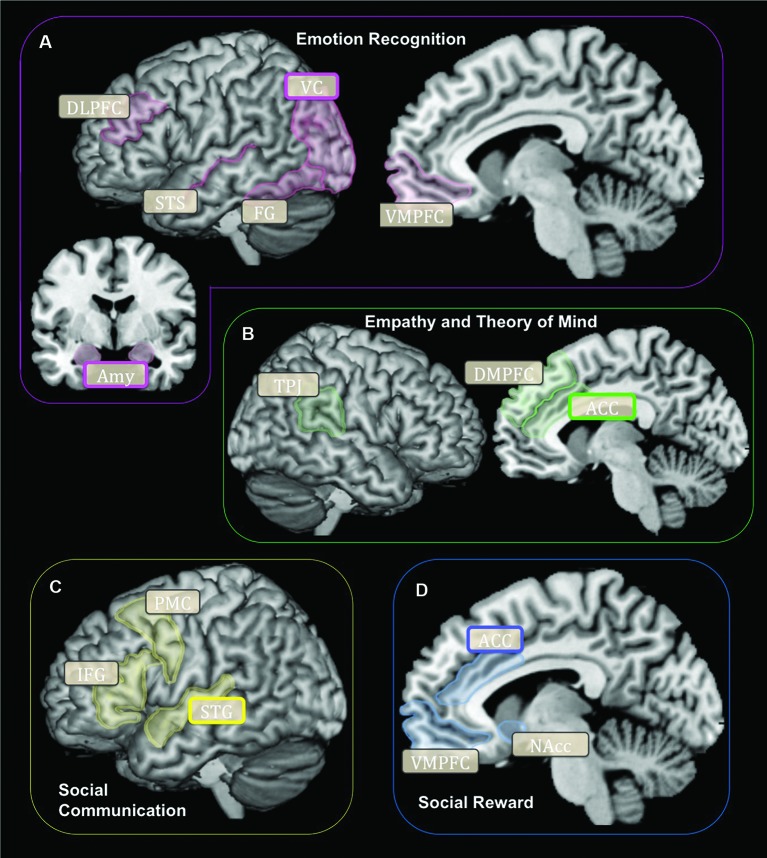
**Schematic representation of brain networks subserving cooperation for each social-cognitive component: (A) Emotion Recognition, (B) Empathy and Theory of Mind, (C) Social Communication, (D) Social Reward**. Rounded boxes (with bold or grey outlines) signify brain regions implicated in social-cognitive constructs subserving cooperation. Rounded boxes with bold outlines signify brain regions shown to be structurally or functionally different according to the *OXTR* gene (SNPs or methylation) in humans. DLPFC, Dorsolateral Prefrontal Cortex; VC, Visual Cortex; STS, Superior Temporal Sulcus; FG, Fusiform Gyrus; VMPFC, Ventromedial Prefrontal Cortex; Amy, Amygdala; TPJ, Temporoparietal Junction; ACC, Anterior Cingulate Cortex; PMC, Premotor Cortex; IFG, Inferior Frontal Gyrus; STG, Superior Temporal Gyrus; NAcc, Nucleus Accumbens.

Neuroimaging research demonstrates that specific brain networks carry out many of the social-cognitive constructs underlying cooperation. The ability to accurately acknowledge and recognize the emotional states of others (emotion recognition) is carried out through the ventral processing stream and relies on visual cortices, the fusiform gyrus (FG), superior temporal sulcus (STS) and areas within the prefrontal cortex (Figure 1A). The visual centers of the brain are within an emotional attention circuit (Rudrauf et al., 2008). When faced with information of high emotional saliency, the amygdala and visual cortex (VC) function to increase local attention recourses (Morris et al., 1998). The FG contains the fusiform face area, which is a highly specialized region for distinguishing between different types of faces (Kanwisher et al., 1997). The amygdala functions to signal and tag information that is highly emotionally salient (Aggleton, 2000). The STS is involved in processing social and emotional signals conveyed via body or biological motions (Thompson and Parasuraman, 2012). Lastly, the ventromedial and dorsolateral prefrontal cortex (DLPFC) categorizes and evaluates the salience of emotional stimuli (Mitchell and Greening, 2012; Roy et al., 2012). Combined, this brain network subserves the ability to evaluate and categorize the emotional states of others.

Being able to accurately interpret the intentions of others (empathy and theory of mind) relies on a brain network that includes areas within the frontal, temporal and parietal cortices (Figure 1B). The dorsomedial prefrontal cortex (DMPFC) and dorsal anterior cingulate cortex (ACC) generate appropriate emotional responses to other people’s mental states (i.e., emotional empathy) (Fan et al., 2010). While the temporoparietal junction (TPJ) is involved in accurately interpreting the mental states and intentions of others (i.e., cognitive empathy or theory of mind) (Decety and Lamm, 2007). Together, these brain structures subserve the ability to accurately understand and respond to the emotions and intentions of other people.

Social communication (the ability to perceive, transmit and understand information between people) relies on brain regions that include several areas within the temporal and frontal lobes (Figure 1C). The superior temporal gyrus (STG) processes verbal and non-verbal social cues (Hickok and Poeppel, 2007; Hein and Knight, 2008). The superior temporal gyrus contains the primary auditory cortex, which functions to decode vocal communication signals (Hickok and Poeppel, 2007). Lastly the inferior frontal gyrus (IFG), and the premotor cortex (PMC) are both important regions involved in speech production (Price, 2012). Together, the integrity of these brain structures is critical for effective social communication to occur.

Being driven towards social interaction and the subjective sense of reward in response to social interaction may be associated with the tendency to cooperate. The subjective motivation towards social interaction is subserved by a brain network involved in salience and reward processing and includes the dopaminergic system and several regions within the ventral striatum and frontal lobe (Haber and Knutson, 2009; Figure 1D). Within the striatum, the nucleus accumbens (NAcc) is a critical area involved in reward processing and is conceptualized as the brain’s “pleasure center.” Within the frontal lobe, the ventromedial prefrontal cortex (VMPFC) and ACC function to signal and anticipate potential rewards (Rushworth et al., 2011). Thus, the brain’s reward circuitry may contribute to how social interactions are experienced and valued during conditions that involve cooperation.

## *OXTR* and brain mechanisms underlying cooperation

In this section, we will consider the association between the *OXTR* gene and specific social-cognitive constructs and brain networks that subserve cooperation. There is some evidence that the *OXTR* gene is associated with emotion recognition. Lucht et al. (2012) and Rodrigues et al. (2009) reported that performance on the “Reading the Mind in the Eyes Test” varied according to *OXTR* polymorphisms. Additional, indirect support for the association between OT genes and emotion recognition comes from evidence that OT administration improves people’s ability to recognize emotions (Domes et al., 2007; Bartz et al., 2010; Guastella et al., 2010).

In terms of the brain, magnetic resonance imaging studies (MRI) studies show that the *OXTR* gene is associated with the structure and function of a subset of brain regions involved in emotion recognition (Figure 1A). O’Connell et al. (2012) demonstrated that *OXTR* polymorphisms are associated with VC (cuneus and inferior occipital gyrus) reactivity to fearful faces. In terms of the amygdala, both Furman et al. (2011) and Inoue et al. (2010) showed an association between the *OXTR* gene and amygdala volume and Tost et al. (2010) showed that the *OXTR* gene is associated with amygdala activity during an emotional face-matching task. For the FG, O’Connell et al. (2012) directly tested for activation differences according to *OXTR* variants within this region, but failed to identify any significant differences. A recent study investigated the association between methylation^1^ of the *OXTR* gene and brain function during biological motion processing (Jack et al., 2012). The results indicated that increased methylation (typically associated with decreased expression) of *OXTR* is associated with greater activation within the superior temporal gyrus (STG). Although biological motion is more often linked with the function of the STS, this study provides preliminary evidence that the *OXTR* gene is associated with neural reactivity during the recognition of social information. Lastly, it is currently unknown if the *OXTR* gene is associated with the structure or function of the dorsolateral or VMPFC in humans.

There is evidence that the *OXTR* gene may be linked to individual differences in empathy and theory of mind. Behavioral studies show that *OXTR* is associated with self reported empathy (Rodrigues et al., 2009). Furthermore, Wu et al. (2012) demonstrated that distinct polymorphisms of the *OXTR* gene are associated with emotional and cognitive (i.e., theory of mind) empathy. In terms of the brain, there is evidence that the *OXTR* gene is associated with the structure and function of brain regions involved in emotional empathy (Figure 1B). Specifically, Furman et al. (2011) and Tost et al. (2010, 2011) demonstrated volumetric differences of the dorsal ACC according to *OXTR* polymorphisms. In terms of function, Tost et al. (2011) showed that the *OXTR* gene is associated with dorsal ACC activity during emotional face processing. However, it is currently unknown if *OXTR* is associated with the structure or function of key brain regions involved in cognitive empathy/theory or mind, such as the temporal parietal junction.

The *OXTR* gene may be linked to the ability to socially communicate. Behavioral research shows that *OXTR* is associated with the ability to comprehend information during vocal communication (Tops et al., 2011) and with the severity of communication deficits in autism (Jacob et al., 2007; Lerer et al., 2007; Campbell et al., 2011). There is limited evidence that *OXTR* is linked to brain mechanisms underlying social communication (Figure 1C). However, indirect support comes from research on the effect of OT administration on brain reactivity to vocal social signals (Riem et al., 2011, 2012). Riem et al. (2011) showed that parents that receive OT administration exhibit greater left inferior frontal gyrus reactivity to sounds of their child crying as compared to parents receiving placebo. In addition, *OXTR* methylation status is associated with left STG activation (Jack et al., 2012). However, the association between *OXTR* methylation and STG activity was found in response to a biological motion processing task. Therefore, it is currently unknown if the *OXTR* gene is associated with the functional anatomy of the STG when socially communicating. In addition, it is currently unknown how *OXTR* polymorphisms may affect the structure or function of other regions involved in social communication that include the inferior frontal gyrus and premotor area.

The *OXTR* gene may be associated with individual differences in social reward processing. Behavioral studies indicate that the *OXTR* gene is associated with trait reward sensitivity (Tost et al., 2010) and social motivation deficits in autism (Campbell et al., 2011). In terms of the brain, there is some evidence that the *OXTR* gene is associated with the structure and function of a subset of regions involved in reward processing (Figure 1D). Polymorphisms of *OXTR* are associated with ACC reactivity during emotion processing (Tost et al., 2011). In addition *OXTR* is associated with volumetric differences of the ACC (Tost et al., 2010; Furman et al., 2011). Indirect support of the association between *OXTR* and the neural basis of reward processing comes from research on the link between *OXTR* and dopamine transmission. Love et al. (2012), used positron emission tomography and showed that *OXTR* is associated with dopamine levels within the striatum in females. Lastly, research on animals demonstrates that the nucleus accumbens is densely populated with OT receptors (Ross et al., 2009). It is currently unknown however, how the *OXTR* gene affects the functional anatomy of the Nacc and VMPFC in humans.

A review of the research to date demonstrates that the *OXTR* gene may influence the functional anatomy of a subset of the brain regions implicated in cooperation. For emotion recognition, the *OXTR* gene is associated with the structure (Inoue et al., 2010; Furman et al., 2011) and function (Tost et al., 2010) of the amygdala and the function of the VC (O’Connell et al., 2012). This indicates that the *OXTR* gene may be associated with attention to emotionally salient stimuli, but not necessarily with face processing (fusiform gryus) or higher order categorization and evaluation of emotional stimuli (VMPFC and DLPFC). For empathy and theory of mind, the *OXTR* gene is associated with the structure (Tost et al., 2010, 2011; Furman et al., 2011) and function (Tost et al., 2011) of the ACC, though there is currently no evidence that *OXTR* is associated with the structure or function of the TPJ. These findings suggest that the *OXTR* gene may influence brain regions involved in emotional but not cognitive empathy. In terms of social communication, there is one study showing an association between *OXTR* methylation and the function of the STG (Jack et al., 2012). Therefore, it is currently not known how *OXTR* polymorphisms are linked to the functional anatomy within brain important for social communication. Lastly, for social reward processing, there is evidence that *OXTR* is associated with the structure (Tost et al., 2010, 2011; Furman et al., 2011) and function (Tost et al., 2011) of the ACC. In spite of many animal studies demonstrating that the NAcc is densely populated with OT receptors (Hammock and Young, 2006), it is currently unknown how the *OXTR* gene may affect the functional anatomy of the NAcc in humans.

## Potential future research directions and conclusion

There is currently a lack of evidence that the *OXTR* gene is associated with brain function during tasks that explicitly involve cooperation. One potential strategy to elucidate the association between *OXTR* and the brain basis of cooperation is to utilize a version of the prisoner’s dilemma task that can be used within a brain imaging environment (Rilling et al., 2012) and compare patterns of brain reactivity according to *OXTR* polymorphisms.

In addition, a potential strategy to explore how the *OXTR* gene may be associated with face processing is to use tasks and analysis procedures specifically designed to quantify the spatial extent of the fusiform face area (Weiner and Grill-Spector, 2012). Support for the hypothesis that the *OXTR* gene is associated with emotional but not cognitive empathy may be obtained by using fMRI tasks designed to explicitly compare types of empathic processing (Sebastian et al., 2012). For social communication, MRI studies show that emotional prosody relies on a specific network of brain regions (Ethofer et al., 2006; Wiethoff et al., 2008). Emotional prosody tasks may be a promising tool to explore the association between *OXTR* and brain networks subserving social communication. Lastly, an effective way to investigate the association between *OXTR* and social reward processing may be to utilize tasks explicitly designed to assess social versus non-social (monetary) reward processing (Gossen et al., 2013). Based on findings that social relevance boosts the influence of OT on cooperative behavior (Declerck et al., 2010, 2013), the *OXTR* gene may have a greater impact on brain function during social reward processing as compared to monetary reward processing.

In this review, we have focused on a network of key brain regions involved in cooperation. In conclusion, there is limited evidence that the *OXTR* gene is directly linked to the functional anatomy of the brain network implicated in cooperation. The use of endophenotypes is a promising strategy that may help to elucidate this complex gene, brain and social-cognitive association.

## Conflict of interest statement

The authors declare that the research was conducted in the absence of any commercial or financial relationships that could be construed as a potential conflict of interest.

## Footnotes

^1^Methylation is an epigenetic process that affects the expression of genes.
